# A Randomised Control Trial Investigating the Efficacy of the MapMe Intervention on Parental Ability to Correctly Categorise Overweight in Their Child and the Impact on Child BMI Z-Score Change at 1 Year

**DOI:** 10.3390/children10091577

**Published:** 2023-09-21

**Authors:** Angela R. Jones, Kay D. Mann, Laura R. Cutler, Mark S. Pearce, Martin J. Tovée, Louisa J. Ells, Vera Araujo-Soares, Bronia Arnott, Julie M. Harris, Ashley J. Adamson

**Affiliations:** 1 Population Health Sciences Institute, Newcastle University, Framlington Place, Newcastle upon Tyne NE2 4HH, UKmark.pearce@newcastle.ac.uk (M.S.P.); bronia.arnott@newcastle.ac.uk (B.A.); ashley.adamson@newcastle.ac.uk (A.J.A.); 2 Human Nutrition and Exercise Research Centre, Newcastle University, Framlington Place, Newcastle upon Tyne NE2 4HH, UK; 3 Health Capital Division, Queensland Health, 33 Charlotte Street, Brisbane, QLD 4000, Australia; kay.mann@health.qld.gov.au; 4 Health and Life Sciences, Northumbria University, Newcastle upon Tyne NE1 8ST, UK; martin.j.tovee@northumbria.ac.uk; 5 School of Clinical & Applied Sciences, Leeds Beckett University, Leeds LS1 3HE, UK; l.ells@leedsbeckett.ac.uk; 6 Center for Preventive Medicine and Digital Health, Medical Faculty Mannheim, Heidelberg University, D-68167 Mannheim, Germany; vera.araujo-soares@medma.uni-heidelberg.de; 7 School of Psychology and Neuroscience, University of St Andrews, St Andrews KY16 9TS, UK; jh81@st-andrews.ac.uk

**Keywords:** children, overweight, obesity, parents, body size perception, weight status, intervention

## Abstract

Research suggests parental ability to recognise when their child has overweight is limited. It is hypothesised that recognition of child overweight/obesity is fundamental to its prevention, acting as a potential barrier to parental action to improve their child’s health-related behaviours and/or help seeking. The purpose of this study was to investigate the efficacy of an intervention (MapMe) to improve parental ability to correctly categorise their child as having overweight one-month post-intervention, and reduce child body mass index (BMI) z-score 12 months post-intervention. MapMe consists of body image scales of known child BMI and information on the consequences of childhood overweight, associated health-related behaviours and sources of support. We conducted a three-arm (paper-based MapMe, web-based MapMe and control) randomised control trial in fifteen English local authority areas with parents/guardians of 4–5- and 10–11-year-old children. Parental categorisation of child weight status was assessed using the question ‘How would you describe your child’s weight at the moment?’ Response options were: underweight, healthy weight, overweight, and very overweight. Child weight status and BMI z-scores were calculated using objectively measured height and weight data and UK90 clinical thresholds. There was no difference in the percentage of parents correctly categorising their child as having overweight/very overweight (*n* = 264: 41% control, 48% web-based, and 43% paper-based, *p* = 0.646). BMI z-scores were significantly reduced for the intervention group at 12 months post-intervention compared to controls (*n* = 338, mean difference in BMI z-score change −0.11 (95% CI −0.202 to −0.020, *p* = 0.017). MapMe was associated with a decrease in BMI z-score 12 months post-intervention, although there was no direct evidence of improved parental ability to correctly categorise child overweight status. Further work is needed to replicate these findings in a larger sample of children, investigate mechanisms of action, and determine the use of MapMe as a public health initiative.

## 1. Introduction

Efforts to curb the international rise in childhood obesity (OB) have failed, with the prevalence of OB in the young increasing globally [1]. The report for the Commission of Ending Childhood Obesity (ECHO) acknowledges that no single intervention can halt the OB epidemic and a multidimensional approach is required [2]. One area for action highlighted by this report is tackling the obesogenic environment and social norms; such norms influence the perception of healthy or desirable body weight, especially in the young [2].

A wealth of data exploring parents’ perceptions of child body weight exists and shows that parents typically do not categorise their child with overweight (OW) as such [3,4,5]. A survey from the WHO European Childhood Obesity Surveillance Initiative reported that most parents seem to be more likely to underrate the body weight of their children if their children have OW/OB [6]. In addition, parents have been found to be shocked and shown disbelief when they are informed of their child’s objectively measured OW status [7]. These findings are important because parents play a key role in the development and maintenance of their child’s health-related behaviours, and in the case of children with OW, seeking and accepting the appropriate support for child weight management [4]. It has therefore been assumed that without recognition of child OW, parents are unlikely to take appropriate action to change their child’s dietary or physical activity behaviours, or seek support to address their child’s weight status [8].

Monitoring of child growth and identification of children with OW is typically completed by health professionals using an appropriate age- and sex-specific growth chart [9]. However, evidence shows that parents lack trust in such measures and tend to use visual assessments and comparisons with peers when determining child weight status [10,11,12]. The use of alternative methods to growth chart-based approaches has the potential to support discussions on children’s growth [13] and weight status with parents. Evaluation of interventions built around the evidence on how parents determine child weight status is necessary to identify how parental categorisation of their child’s OW status can be improved.

As parents use more visual assessments to monitor their child’s weight, a visual tool may help them to understand what a child with OW looks like and in turn support them to prevent or address unhealthy weight gain in their child. Body image scales (BIS) are visual images of body shapes of different sizes, and Jones et al. [14] have reported the development of BIS of known weight status for 4–5- and 10–11-year-old children, according to British growth reference (UK90) clinical thresholds [15]. The BIS are one feature of an intervention (MapMe). MapMe also includes information on childhood OW, healthy eating, physical activity and sources of support. The aims of this study were to examine the efficacy of the MapMe intervention on parental ability to correctly categorise their child as having OW one-month post-intervention, and to determine the longer-term impact on child body mass index (BMI) z-scores at 12 months post-intervention.

## 2. Materials and Methods

### 2.1. Participants

Parents of 4–5- and 10–11-year-old children were recruited to the 4 & Understanding Parental Perceptions Study (4 & UPP Study): a randomised control trial testing an intervention (MapMe) designed to improve parental ability to correctly categorise childhood OW and knowledge of its consequences. Parents gave written consent for their own and their child’s participation in this study. Older children also gave written assent for their participation.

### 2.2. Procedure

**Figure 1:**
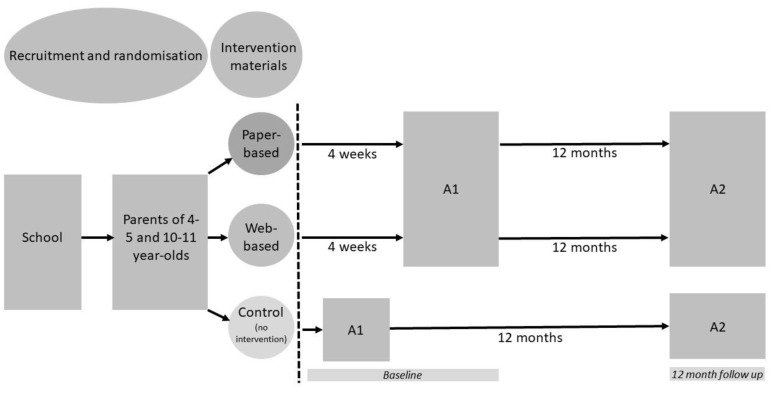
Study design. A1 = Assessment 1, A2 = Assessment 2. Child height and weight measured by National Child Measurement Programme at baseline, and by the study team at 12 months follow up. Paper-based = parents were provided with age- and sex-specific paper-based body image scales and an information booklet on the consequences of childhood overweight, health related behaviours and sources of support. Web-based = parents were provided with age- and sex- specific body image scales in 3D which could be rotated 360°. Parents were also shown, in 3D, what their child may look like as a young adult should they stay within the same weight category. Parents were then signposted to information on health related behaviours and sources of support including online resources tailored to the weight status of the child.

Parents of 4–5- and 10–11-year-old children were recruited via schools or local authority mail out. Randomisation of parents to one of the three study arms (paper-based or web-based MapMe intervention or control (no intervention)) occurred at the school level (see below). The primary mode for providing parents with their intervention materials (whether paper-based or web-based) was via post. Those parents randomly assigned to the web-based arm, who provided an email address, also received a follow-up email with the website link. Four weeks after the provision of the intervention materials all parents in an intervention arm were posted the study questionnaire (‘Assessment 1’—see Figure 1 for study design). The study questionnaire included an assessment of parental categorisation of their child’s weight status. Parents allocated to the control arm provided baseline data for the trial; they did not receive any intervention materials and were asked to complete the study questionnaire via post soon after consent [16].

To assess parental ability to correctly categorise their child’s weight status, an objective measure of child weight status was needed. With parent permission, National Child Measurement Programme (NCMP) data were obtained for the children. The NCMP is a programme in England which monitors OW and OB prevalence in 4–5- and 10–11-year-old children nationally by measuring children’s height and weight within the school environment [17]. Where these data were not available, the study team completed height and weight measurements either at school or at home. 

All children were followed up after 12 months (‘Assessment 2′—Figure 1). Their height and weight were measured either at school or home by the study team following the NCMP protocol. Parents were also posted the follow-up study questionnaire. 

A favourable ethical opinion for this study was obtained from the National Research Ethics Service Committee North East—Newcastle & North Tyneside 2: reference 12/NE/0409. Trial registration reference ISRCTN91136472.

### 2.3. Eligibility and Randomisation

In order to link with the NCMP, schools were drawn from local authority areas within England. Special needs schools or fee-paying schools were ineligible for this study since the NCMP involves mainstream state-maintained schools, including academies, only. Since the MapMe BIS were developed using a largely White ethnic population [14], schools with >35% Black or minority ethnic populations were also ineligible.

Schools involved in this study were stratified into low, medium and high Index of Multiple Deprivation (IMD, 2010) tertiles (based on school postcode) by the study team. IMD is a commonly used indicator of deprivation, it is used by the NCMP and combines a number of indicators which cover a range of economic, social and housing issues into a single deprivation score for each small area in England [18]. The stratified school data were provided to the Newcastle Clinical Trials Unit and a statistician, independent to the study team, randomly allocated each of the schools to one of the study arms.

### 2.4. The MapMe Intervention

MapMe comprised of age- and sex-specific BIS, the development of which have been described elsewhere [14], and supporting information on childhood OW, healthy eating, physical activity and sources of support. The BIS contains photorealistic and anthropometrically accurate illustrations of children aged 4–5 and 10–11 based on 3D scans of hundreds of children [14]. It was made available in two formats; paper based and web-based (see below for further details on the two formats). The purpose of the BIS was to re-calibrate parents’ weight status decision boundaries, countering effects of visual normalisation of childhood OW [19] and perceptual biases [20] in weight status, and so improve parents’ ability to correctly categorise child weight status. The aim of the supporting information was to improve parents’ knowledge of the consequences of childhood OW as well as provide information on healthy eating, physical activity and sources of support. The supporting information signposted parents to sources of information and professional support, which included motivational and volitional materials for goal setting, practice, and action and coping planning in family-based dietary and activity changes [21,22]. The intervention was informed by Social Cognitive Theory [23], which proposes, in terms of behavioural capability, that in order to perform a given behaviour, both the necessary skill(s) and knowledge of why it is important are needed, providing one without the other is not sufficient. Thus, MapMe addressed the skills of parents to categorise an unhealthy weight in their child, the understanding of the health risks associated with unhealthy weight gain in childhood, and strategies to target the issue to facilitate parent behaviour change. The focus was on parental behaviour change since parents are the key agents for change in child weight management at these younger ages [24].

Parents within the paper-based intervention arm were provided with the MapMe intervention with the BIS relevant to their child (i.e., specific to their child’s age and sex). They were asked to view the images and choose the image most like their child before being informed on the following page of the weight status of each body image. Parents in this arm were also provided with an information booklet detailing the consequences of childhood OW and information on healthy eating, physical activity and sources of support. Parents in the web-based intervention arm were given details of the password protected study website and instructions on how to access. The web-based format of MapMe provided parents with the MapMe BIS appropriate for their child in 3D. Parents were able to rotate the BIS images 360° and were asked to choose the image which looked most like their child. Parents were then asked to enter their child’s height and weight details, following this were shown the image and weight status that corresponded to that data (i.e., they were informed whether their choice of image was ‘correct’ or not), thus facilitating parental acknowledgment of their child’s weight status. A 3D image of a generic young adult appropriate to the child’s sex was then shown in order to indicate to parents what their child may look like as a young adult should they stay within the same weight status category. This was accompanied by information on the health-related risks associated with childhood OW, tapping into parental concerns of future OW in their child [12] and raising awareness of potential health consequences. Parents were then signposted to sources of information regarding healthy eating, physical activity and sources of support including online resources tailored to the weight status of the child.

### 2.5. Measures

*Parents’ categorisation of their child’s weight status*: Parents’ categorisation of their child’s weight status was assessed using a categorical questionnaire item: ‘How would you describe your child’s weight at the moment?’. The response options were: underweight; healthy weight; overweight; very overweight. As described above, parents allocated to the control arm provided baseline data for the trial and were asked to complete the study questionnaire via post soon after they consented to participate. Parents allocated to the intervention arms were asked to complete the study questionnaire four weeks after the provision of the intervention materials (Figure 1).

*Child anthropometric measures*: The majority of child height and weight measurements at baseline were completed by NCMP teams. When necessary at baseline, and for each child at 12 months follow up, anthropometric data were collected by the study team as follows: Height was measured to 0.1 cm using a Leicester portable height measure (Chasmors, London, UK) with the head in the Frankfort plane and weight was measured to 0.1 kg in light indoor clothing using Shekel scales (Shekel Scales Ltd., Nof Hagalil, Israel). Measurements were taken at least in duplicate until two values were obtained within 1.0 cm of each other for height and within 0.1 kg of each other for weight, the mean for each measure was used in the analyses.

*Socioeconomic status*: The postcode of each family’s address at recruitment was used to assign the 2010 IMD quintile.

*Parents’ education*: Parents’ level of education was assessed at follow up using a categorical questionnaire item: Please look at the list below and state your highest certified educational qualification. The response options were: none; GCSE or equivalent; A level or equivalent; higher BTEC or equivalent; University/College degree.

*Patient and public involvement*: A panel of parents and a panel of health professionals were consulted throughout the development of the BIS [14] and MapMe intervention. The Trial Steering Committee included a parent member. Small-scale feasibility testing completed prior to the main trial also gathered parent views and opinions on the study materials and the feedback enabled us to make alterations to them as appropriate.

### 2.6. Data Analysis

Baseline data were collected between March 2013 and July 2014. Follow-up data were collected between May 2014 and November 2015. Child height and weight measurements were used to calculate BMI (weight (kg) divided by height (m^2^)). From BMI, each child’s age and sex-specific BMI z-score and weight status category was identified using UK90 clinical thresholds, OW was defined as ≥91st and <98th centile (≥+1.33 and <+2.00 z-score) and very OW (OB) was defined as ≥98th centile (≥+2.00 z-score) [15]. Using objective child weight status data and parental categorisation data, accuracy of parental categorisation was determined as ‘correct’ or ‘incorrect’. Parents were deemed ‘correct’ if their child was underweight and categorised as underweight and if their child was healthy weight and categorised as healthy weight. Parents were also deemed ‘correct’ if their child had OW and was perceived as having OW or very OW and if their child had very OW and was perceived as having OW or very OW, i.e., parents identified some level of OW in their child.

Analyses examining the efficacy of the MapMe intervention on parental ability to correctly categorise OW in their child included those families for whom: child height and weight measures had been obtained at baseline, and from these measurements the child had been identified as having OW or very OW; and the measure of parental categorisation of their child’s weight status had been completed via the study questionnaire. Comparisons of parental accuracy in their categorisation of their child’s weight status between study arms, and the two intervention arms combined versus the control arm, were assessed using chi-squared tests. Multivariable logistic regression was used to assess predictors of parental ability to correctly categorise their child as having OW/very OW. Analyses examining the efficacy of the MapMe intervention on child weight change at 12 months post-intervention included only those children who were identified as having OW or very OW at baseline for whom measures of height and weight at 12 months follow up were available.

SPSS v.24 was used to complete all data analysis. Significance was set at *p* < 0.05.

## 3. Results

### 3.1. Recruitment and Demographic Data

A total of 36 980 families from 15 local authorities across England were invited to take part in the 4 & UPP study. A total of 2933 parent–child pairs were recruited (7.9% consent rate), and valid height and weight data were obtained for 2765 (94.3%) children. A total of 94% were measured by NCMP teams, and the remainder were measured by the study team. Of those children, 429 (15.5%) were identified as having OW or very OW. Of these 429 children, 53.6% were boys and 36.6% were aged 4–5 years (Table 1). The families were from a range of socioeconomic groups although there was a smaller percentage of children with OW from the least deprived IMD quintiles compared to the most deprived IMD quintile. Most parents recruited were mothers (90.9%, Table 1). Of the 429 children with OW or very OW, 267 (62.2%) of their parents returned the study questionnaire and provided their categorisation of their child’s weight status. A total of 225 (52.4%) of their parents indicated their highest certified educational qualification, with most (*n* = 104; 46.2%) being educated to University/College degree level. At follow up, children were measured a mean of 13.6 months after their baseline measurement (range 7.0–26.1; median 13.5); 71.2% were re-measured 12 +/− 3 months after their baseline measurement. All measures were reported as age (at time of measurement) and sex-specific BMI z-score. A total of 338 (78.8%) of the children identified as having OW or very OW at baseline were measured at follow up.

### 3.2. Parents’ Categorisation of Their Child’s Weight Status

Of the sample of 267 parents, 116 (43.4%) correctly categorised their child as having OW or very OW. The majority of children with OW were categorised by their parents as having a healthy weight (*n* = 121, 72.9%; 26.5% (*n* = 44) were categorised as having OW), and the majority of children with very OW were categorised as having OW (*n* = 66, 65.3%; 5.9% (*n* = 6) were categorised as having very OW).

Provision of the MapMe intervention was not significantly related to whether the parents correctly categorised their child’s weight status (41% control, 45% all intervention, *p* = 0.506). No significant differences were observed when examining the intervention arms separately (41% control, 47% web-based intervention, 44% paper-based intervention, *p* = 0.750, Table 2).

Multivariable logistic regression (*n* = 267) examining predictors of parental ability to correctly categorise their child’s OW/very OW status, showed that the odds of a parent correctly categorising their child with OW/very OW as such, were significantly increased if the child was a girl (odds ratio = 2.4, 95% confidence interval (CI) 1.4 to 4.0, *p* = 0.002) and in the older age group (odds ratio = 5.3, 95% CI 3.0 to 9.5, *p* < 0.001). There was no interaction between sex and age. 

### 3.3. Child Weight Change 12 Months Post-Intervention

At the 12-month follow up, height and weight measurements were obtained for 338 (78.8%) of the children who were identified as having OW or very OW at baseline. A total of 92 (27.2%) of these children had an improved weight status after a year (for example, children with very OW became children with OW or healthy weight, Chi^2^ = 110.2, *p* < 0.001).

Table 3 shows the mean change in BMI z-score between baseline and follow up for those children identified as having OW or very OW at baseline. Mean BMI z-score at follow up was lower across all arms of this study; however, this was significant only in the intervention arms (Table 3). When assessing differences in the change between study arms, the mean difference in BMI z-score change between those in any intervention arm and the control arm was −0.11 (95% CI −0.202 to −0.020, *p* = 0.017). The mean difference in BMI z-score change was −0.13 (95% CI −0.237 to −0.027, *p* = 0.014) and −0.09 (95% CI −0.197 to 0.015, *p* = 0.091) between the Web-based intervention and Paper-based intervention arms and control arm, respectively. Differences between the two intervention arms were not significant (−0.04, 95% CI −0.150 to 0.068, *p* = 0.525).

## 4. Discussion

Most parents of children with OW or very OW underestimated their child’s weight status category, regardless of study arm. Being provided with the MapMe intervention, however, was associated with a reduced BMI z-score at 12 months post-intervention.

Our findings on the percentage of parents correctly categorising their child’s OW or very OW status are consistent with previous research [3,4,5]. Similarly, our observations that child age and sex were important factors in parental identification of childhood OW are consistent with previous research [3,5,25].

Despite the wealth of data available on the limited ability of parents to identify when their child has OW/very OW and suggestions that this is a key issue to address [3], few studies have directly examined the issue of how parental recognition can be improved and whether attempts to improve recognition lead to positive child weight outcomes in the long term. Rune et al. [26] examined the effectiveness of an educational intervention which included an information leaflet and colour-coded BMI charts in improving parental perception of child weight. The intervention had no impact on accuracy of parents’ perception of their child’s weight status [26]. Pakpour et al. [27] also tested the impact of an educational intervention on recognition of childhood OB via a series of lectures and a leaflet on childhood OB, growth charts and how to measure BMI in children. The intervention was found to significantly improve mothers’ ability to identify childhood OB [27]; however, the intervention would be both labour intensive and expensive, and so may not be feasible for implementation at a wider population level. Neither Rune et al. [26] nor Pakpour et al. [27] examined child weight outcomes post-intervention. Perrin et al. [28] tested an intervention for paediatricians to prevent and treat OW and OB which included colour-coded BMI charts, and a nutrition and activity-focused assessment and counselling tool. The percentage of parents accurately assessing their child as having OW at 1- and 3- month follow-up clinic visits improved; however, significant improvements from baseline were only observed at 3 months. Changes in weight status were examined at both follow-up visits but were not statistically significant [28]. The short-term nature of the follow-up periods limited the ability to examine weight trajectories over the longer term.

In the present study, the longer-term impact of the intervention on child weight outcomes was examined and significant reductions in mean BMI z-score were observed in those children with OW/very OW in the intervention arms at 12 months follow up. The mean change of −0.12 is approximately half that observed in a study of children with OB who completed an intensive community based intervention which invited participants to attend eighteen group sessions and provided families with a free swimming pass for 12 weeks (−0.23, *p* < 0.0001) [29]. The mean BMI z-score change seen in the intervention arms in this study also compares favourably with that found in a Cochrane review of 70 randomised controlled trials examining the effects of diet, physical activity and behavioural interventions for the treatment of OW and OB in 6–11-year-old children (−0.06, *p* = 0.001) [30].

Importantly, the MapMe intervention was associated with positive weight outcomes in children with OW/very OW 12 months post-intervention despite there being no evidence of a similar effect on parental ability to classify childhood OW. This finding seems contrary to our theoretical approach which suggested that in order to facilitate parent behaviour change parents needed both the skill of being able to correctly categorise their child as having OW, and the knowledge of why it is important; however, this finding may be due to lack of power to detect this change.

Strengths of this study are that it was a large randomised control trial involving families from 15 regions across England. The intervention (in both formats) was minimally invasive and inexpensive to deliver, and so has potential for wider utility. This trial also included a 12-month follow up and demonstrated good retention (79%). Conversely, the consent rate was low (8%), with an under-representation of children with OW/very OW (16%), highlighting the difficulties in engaging families of unhealthy weight children in this area of research, and limiting power in the analyses. Furthermore, the BIS used in this trial were developed using a largely White ethnic population [14]. Given that different ethnic groups have different proportions of fat to muscle for a given BMI and different patterns of fat deposition [31,32], children of different ethnic backgrounds will have different body sizes and shapes for a given BMI. So, although the sets of bodies used in this study are anthropometrically accurate illustrations of BMI change in a White population, they may have a more limited applicability to other ethnic groups. In addition, there were no objective measures of weight-related health behaviours (food intake or physical activity).

## 5. Conclusions and Future Research

To our knowledge, this is one of the first studies to examine the efficacy of an intervention (MapMe), which included BIS of known weight status, on parental ability to correctly identify OW/very OW in their child and the longer-term impact on child BMI outcomes. Although MapMe was not associated with improvements in parental ability to correctly categorise their child with OW/very OW as such, it was associated with improved BMI z-score outcomes at 12 months post-intervention. During the course of the present study, evidence began to emerge suggesting that accurate parental perceptions of their child’s OW/OB does not have a beneficial impact on later weight outcomes; and in some instances, detrimental outcomes have been reported [33,34,35,36]. This study and more recent findings, therefore, suggest that future research could focus on improving parental *acknowledgement* of their child’s weight status and subsequent action rather than reported *perception*.

Future work could also focus on intervention development and examining potential mechanisms of action. Since the BIS in the present study were based on scans of a largely White ethnic population, similar BIS could be developed for other ethnic groups. Given that the present study found that the MapMe intervention, particularly in web-based format, could help weight outcomes of those children with OW/very OW future studies could usefully incorporate comprehensive web-analytics to explore user journey and further add to the evidence base around the impact of e-Health interventions. In this study, this low-cost intervention delivered directly to parents has potential for population level intervention. Application of MapMe as an adjunct to an individual family intervention by a health professional-led tool is worthy of future investigation.

## Figures and Tables

**Table 1:** Demographic data for those children recruited with overweight and very overweight.

	All Children *n* = 429	4–5 Year Olds *n* = 157 (36.6%)	10–11 Year Olds *n* = 272 (63.4%)
*n* (%) boys	230 (53.6)	83 (52.9)	147 (54.0)
*n* (%) overweight	258 (60.1)	104 (66.2)	154 (56.6)
*n* (%) very overweight	171 (39.9)	53 (33.8)	118 (43.4)
BMI Z score IQR	1.55–2.25	1.48–2.16	1.58–2.31
*n* (%) IMD quintile 1	117 (27.3)	35 (22.3)	82 (30.1)
*n* (%) IMD quintile 2	106 (24.7)	38 (24.2)	68 (25.0)
*n* (%) IMD quintile 3	89 (20.7)	33 (21.0)	56 (20.6)
*n* (%) IMD quintile 4	69 (16.1)	30 (19.1)	39 (14.3)
*n* (%) IMD quintile 5	48 (11.2)	21 (13.4)	27 (9.9)
*n* (%) parent recruited is the mother	390 (90.9)	146 (93.0)	244 (89.7)

BMI, body mass index; IQR, interquartile range; IMD, Indices of Multiple Deprivation (1 is most deprived, 5 is least deprived). Overweight/very overweight status was determined using UK90 criteria [15].

**Table 2:** Parental accuracy of their categorisation of their overweight/very overweight child’s weight status.

Study Group	Parental Categorisation	All Children *n* = 267 *n* (%)	Age 4–5 *n* = 106 *n* (%)	Age 10–11 *n* = 161 *n* (%)	Girls *n* = 127 *n* (%)	Boys *n* = 140 *n* (%)
Control						
	*Correct*	46 (41.1)	9 (21.4)	37 (52.9)	28 (56.0)	18 (29.0)
	*Incorrect*	66 (58.9)	33 (78.6)	33 (47.1)	22 (44.0)	44 (71.0)
All intervention						
	*Correct*	70 (45.2)	14 (21.9)	56 (61.5)	39 (50.6)	31 (39.7)
	*Incorrect*	85 (54.8)	50 (78.1)	35 (38.5)	38 (49.4)	47 (60.3)
Web-based intervention						
					
	*Correct*	35 (46.7)	8 (25.0)	27 (62.8)	21 (53.8)	14 (38.9)
	*Incorrect*	40 (53.3)	24 (75.0)	16 (37.2)	18 (46.2)	22 (61.1)
Paper-based intervention						
					
	*Correct*	35 (43.8)	6 (18.8)	29 (60.4)	18 (47.4)	17 (40.5)
	*Incorrect*	45 (56.3)	26 (81.3)	19 (39.6)	20 (52.6)	25 (59.5)

**Table 3:** Mean change in BMI z-score in children with overweight and very overweight between baseline and follow up.

Study Group	*n*	BMI Z-Score			
		Baseline Mean (SD)	Follow-Up Mean (SD)	Change Mean (SD)	*p*-Value *
Control	117	1.89 (0.45)	1.88 (0.56)	−0.01 (0.39)	0.876
All Intervention	221	1.96 (0.49)	1.84 (0.64)	−0.12 (0.41)	<0.001
Web-based intervention	107	2.03 (0.52)	1.89 (0.71)	−0.14 (0.40)	0.001
Paper-based intervention	114	1.89 (0.44)	1.80 (0.57)	−0.10 (0.42)	0.016

BMI, body mass index. * Paired *t*-test.

## Data Availability

Data will be available from the corresponding author on request.
